# HTA responsiveness to today’s challenges to health systems: a responsible innovation in health perspective

**DOI:** 10.1017/S0266462325000121

**Published:** 2025-03-03

**Authors:** P. Lehoux, I. Ganache, O. Demers-Payette, H.P. Silva, G. Plamondon, M. de Guise

**Affiliations:** 1 Institut National d’Excellence en Santé et Services Sociaux (INESSS), Quebec, Canada; 2 Department of Health Management, Evaluation and Policy, University of Montreal; Public Health Research Center (CReSP), 2021 Union Street, Suite 1200, 12th floor, Montreal, Quebec H3A 2S9, Canada

**Keywords:** innovation pathway, health systems, equity, sustainability, climate change, responsible research and innovation, value-based assessment

## Abstract

**Introduction:**

Though Health Technology Assessment (HTA) has steadily grown over the past decades, less attention has been paid to the way HTA may prove more responsive to the broader economic, social, and environmental challenges that health systems are facing today. In view of climate change, chronic diseases, an aging population, inequalities, and workforce issues, the HTA community’s unique set of skills nonetheless holds great potential to help decision-makers strengthen many publicly funded health systems around the world.

**Methods:**

This article adopts an integrated system-wide perspective guided by the Responsible Innovation in Health (RIH) framework to explore how the HTA community may not only adapt to the *speed* of innovation but also consider its direction.

**Results:**

Because RIH aims to steer innovation toward a more sustainable pathway, it can help HTA agencies anticipate decision-makers’ informational needs regarding four systemic challenges: (1) equitable access; (2) workforce issues; (3) accountable policy trade-offs; and (4) environmental sustainability. We clarify how key elements of the RIH framework may be used by HTA agencies to: (1) supplement their evaluation process; (2) align their priority-setting or strategic planning activities with their health system challenges; or (3) inform the production of early HTAs, horizon scans, or reports that are broader in scope than a single technology review.

**Conclusions:**

The article concludes with three practical implications that were identified by the *Institut National d’Excellence en Santé et Services Sociaux* (INESSS) (Québec, Canada) and may inspire other HTA agencies.

## Introduction

The field of Health Technology Assessment (HTA) has grown tremendously over the past decades. It has steadily improved its evidence synthesis methods (1), developed deliberative mechanisms to gather the perspectives of patients and citizens (2), consolidated HTA agencies’ capacities to address ethical and social issues (3), and established more consistent knowledge mobilization strategies to respond to decision-makers’ informational needs (4). During this period rich in novel scientific advances, a steadily growing number of more complex innovative technologies, including drugs, diagnostic tests, and service delivery models, were introduced into health systems. Such rapid technological change complexified HTA’s evidence synthesis processes and increased pressure to deliver guidance to decision-makers in short timeframes. As a result, some HTA scholars warned against the perils of evidence quality deterioration in HTA (5), while others proposed new sophisticated methods to generate more timely evidence such as artificial intelligence-driven syntheses (6), real-world evidence (7), or early health economic modeling (8). However, less attention has been paid to the way HTA may prove more responsive to the broader systemic challenges that health systems are facing today as a result of the cumulated technological, economic, social, and environmental changes that took place since the 1990s (e.g., the American Bayh-Dhole Act translated into increased commercialization of governmentally funded Research & Development activities) (9).

The aim of this article is to help bridge this gap by providing food for thought on what HTA can do in the face of today’s challenges to health systems. One of its key premises is that HTA should not only adapt to the *speed* of innovation but also anticipate its *direction* (10). This entails recognizing the *pathway* health technologies are carving out for health systems. As the latter keep struggling with health inequalities, chronic diseases, an aging population, climate change, and workforce shortages altogether, the current supply-driven technological pathway tends to make health systems increasingly unsustainable (11;12).

To explore the unique role HTA could play in informing decision-makers about the sustainability of health systems, we first clarify how a system-wide perspective highlights four challenges that are exacerbated by the current technological pathway: (i) equitable access; (ii) workforce issues; (iii) accountable policy trade-offs; and (iv) environmental sustainability. We then introduce the Responsible Innovation in Health (RIH) framework (13). RIH is complementary to HTA since it aims to better align the supply of new health technologies with today’s demand for equitable as well as economically and environmentally sustainable solutions (14). Such solutions are needed for publicly funded health systems to remain successful (15) and “true to their mission and values” (16). The RIH framework is used to discuss how the HTA community can anticipate and better address decision-makers’ informational needs regarding the four systemic challenges identified earlier. We conclude with three practical implications that were identified at the *Institut National d’Excellence en Santé et Services Sociaux* (INESSS), Quebec’s HTA agency (Canada), and may inspire other HTA agencies.

## A system-wide perspective for HTA

According to O’Rourke, Werkö (17), the “pipelines of promising, disruptive, and costly innovations” create a steady “demand for more rapid, complex, and broader technology assessments.” One key difficulty for HTA bodies in responding to such demand lies with the need for an integrated policy understanding of health systems, one that acknowledges the complex ways in which new health technologies and health system components interact (16). The dynamic health system framework of van Olmen et al. (18) is a good starting point because it examines how governance, service delivery, human resources, finances, and infrastructures evolve according to shifting contextual factors, including population, knowledge, and values. For instance, a more diversified population (because of migration, aging, or cultural and socioeconomic change) may require a complex mix of chronic and acute care services as well as evidence of these patient groups’ varying needs and health outcomes, which could then inform a value-based (re)allocation of human and financial resources (19).

Likewise, a system-wide perspective can help HTA agencies develop a holistic understanding of the evolving needs and challenges of a health system and the policy issues raised by new health technologies. Though HTA agencies do not develop health policies, they provide guidance to policymakers on a regular basis and can identify the value of technologies that are *responsive* to the challenges affecting the health and social care system in which they operate (20).

### Four key challenges to health systems arising from a supply-driven technological pathway

Health policy research shows that a combination of high cost, poor performance, low-quality care, and inefficiencies are observed in many health systems around the world (21;22). Though multiple factors are at play and vary from one context to another, failings are “especially notable with respect to chronically ill patients, who account for a large fraction of health expenses” while a lack of prevention and “coordinated care that could keep such patients out of the hospital” remains “a key driver of health system inefficiency” (15). These failings result in large part from the way new technologies contribute to exacerbate system-wide challenges rather than to strengthen health systems (16).

Since the 1990s, a supply-driven technological pathway has led to powerful and costly clinical tools including, for instance, medical imaging systems, robotic surgery, and predictive biomarker-based assay platforms (9). However, most of these advances cumulate today into complex policy tensions: they push health systems toward increasingly more service-intensive care that requires high-tech infrastructures and a range of skilled personnel that is mostly found in tertiary care hospitals, thereby widening health outcomes disparities and health inequalities (20).

**Figure 1.**
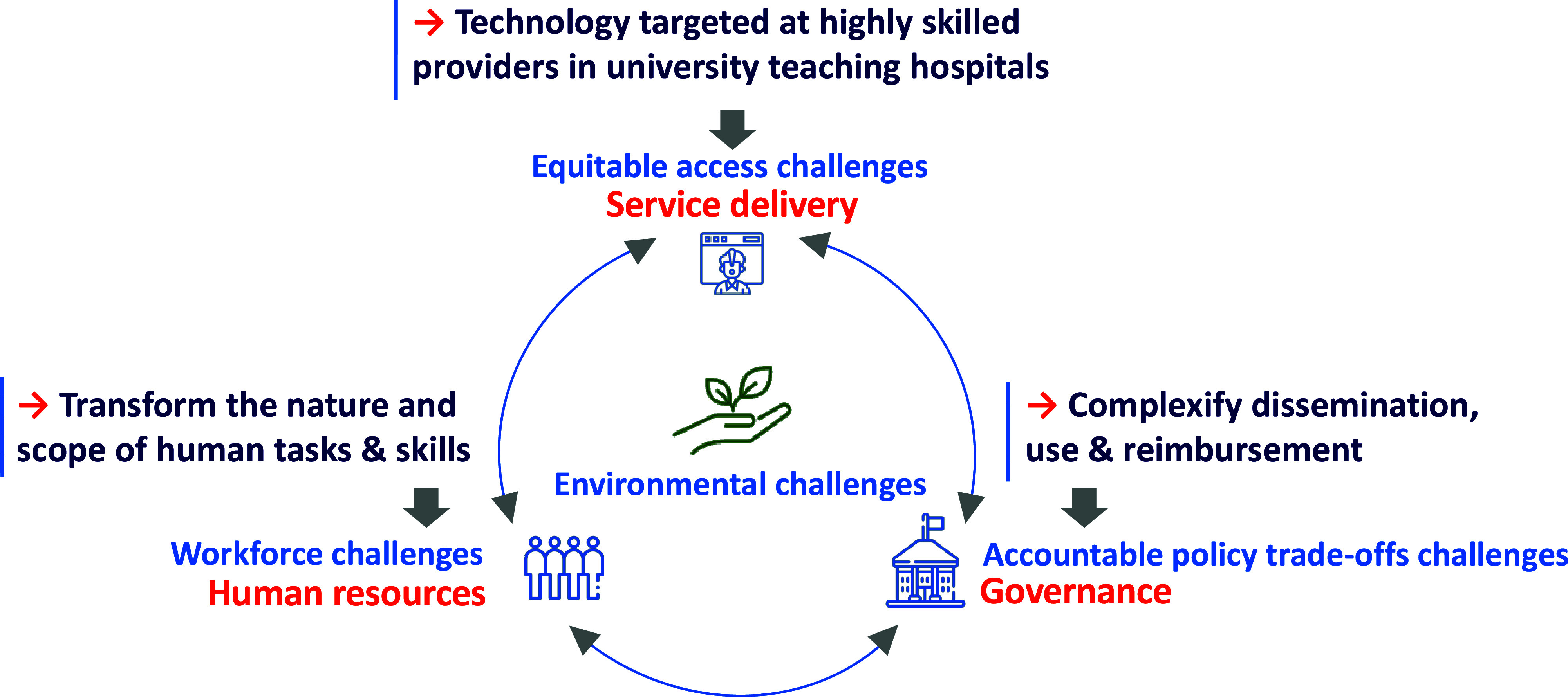
The direction taken by new technologies tends to exacerbate today’s key challenges to health systems. *Source*: the authors.

This technological pathway inadvertently makes a health system less sustainable, as illustrated in Figure 1. Three broad challenges in this figure were identified in an extensive scoping review. Roncarolo, Boivin (23) extracted from 292 scientific articles (published between January 2000 and April 2016 and covering ninety-nine countries) a total of 1590 descriptions of systemic challenges and classified them using the framework of van Olmen et al. (18). The most frequently reported challenges pertained to: (i) governance (21.2 percent); (ii) service delivery (23.8 percent); and (iii) human resources (22.3 percent). The fourth challenge, climate change, has been systematically highlighted in “*The Lancet Countdown*” reports on health and climate change since 2016 (24) and is increasingly attracting the attention of HTA bodies (25-27).

Today’s consequences of a supply-driven technological pathway can be summarized as follows. First, many new technologies target medical specialists as their key users, offering them tools to further develop specialized care areas (e.g., radiology, cardiology, and oncology) (9). The demand for these highly skilled professionals is often outpacing their availability, especially in publicly funded health systems and/or in geographic areas where access to tertiary care hospitals is limited (28). The key issue at play is “how to provide equitable and affordable access to highly specialized care?”

Second, technologies meant to be used by medical specialists often have “corollary” effects as they transform the nature and scope of the skills required by other staff to operate them safely and effectively: biomedical engineers, information system specialists, qualified nurses, technicians, and so forth must be adequately and continuously supported to keep up with technological updates (29). This exacerbates many challenges related to human resources, from staff recruitment, distribution, and retention to the loss of skills associated with turnover (20).

Third, for various clinical, ethical, social, and economic reasons, some technologies are making the proper governance of health systems increasingly more challenging. For instance, gene therapies that come with a two million US dollar price tag per treatment per patient raise daunting policy challenges (30). While experts in the gene therapy field recognize that efficiency in manufacturing and clinical delivery “has always been one of the most, if not the most, formidable problem” (31), such high-profile technologies make it extremely difficult to arrive at consistent and accountable reimbursement decisions (32).

Fourth, the need to reduce the environmental harms caused by health systems is now clearly recognized in part because medical devices account for 13.2 percent of greenhouse gas (GHG) emissions in the health sector (33) and because such harms translate into preventable health problems. For instance, with a health system that is responsible for 4.6 percent of its national GHG emissions, Canada ranks second in per capita health system emissions globally (24), and these emissions may represent “tens of thousands of lost DALYs per year” (34). While definitions of sustainability in health systems emphasize different aspects (see Table 1), a triple bottom-line perspective suggests that a sustainable health system “must adequately deliver across financial, social and environmental concerns” (14) and “do not result in unfair or disproportionate impacts on any significant contributory element of the healthcare system,” including prevention (12).

**Table 1.** Definitions that emphasize different aspects of sustainable health systems. Source: adapted from Zurynski, Herkes-Deane (14) and updated with personal searches by the authors

Focus	Definitions
**Financing**	Fiscal sustainability should be seen as “a requirement, rather than an objective, of health financing policy. Sustainability of healthcare financing therefore cannot be interpreted as a reduction of healthcare costs, but rather as a predictable growth or control of health expenditures” (62).
**Delivery**	Getting human resources “policy and management ‘right’ has to be at the core of any sustainable solution to health system performance” (63). “Ensuring that sufficient resources are available over the long term to provide timely access to quality services that address Canadians’ evolving health needs” (64). A health system “should be built to last, and able to adapt and endure, ensuring that resources are expended efficiently and responsibly to maintain or improve individual and population health and well-being” (14).
**Affordability, acceptability, and adaptability**	A sustainable health system “has three key attributes: affordability, for patients and families, employers, and the government (recognizing that employers and the government ultimately rely on individuals as consumers, employees, and taxpayers for their resources); acceptability to key constituents, including patients and health professionals; and adaptability, because health and health care needs are not static (i.e., a health system must respond adaptively to new diseases, changing demographics, scientific discoveries, and dynamic technologies to remain viable)” (15).
**Planetary health**	“A complex system of interacting approaches to the restoration, management, and optimization of human health that have an ecological base, that is, environmentally, economically, and socially viable indefinitely, that work harmoniously both with the human body and the nonhuman environment, and which do not result in unfair or disproportionate impacts on any significant contributory element of the healthcare system” (12).
**Triple bottom line**	A sustainable health system “must adequately deliver across financial, social, and environmental concerns” (14).

In principle, a stronger command of the key challenges to health systems should help to redefine the direction health technologies should take to strengthen them (20). In practice, there are no specific public organizations responsible for identifying what technologies prove more responsive to health systems’ challenges. HTA is thus in a unique position to generate the multifaceted evidence many policymakers need for health systems to remain both successful and sustainable (15). In a publicly funded health system, this implies appraising whether and how technologies are at the *service* of all its key components and the value they may bring to (or subtract from) the fulfillment of its mission.

## Systemic challenges through the lens of responsible innovation in health

To shed light on the way HTA may respond to decision-makers’ system-level informational needs, we draw on Responsible Innovation in Health (RIH) (13). RIH is a policy-oriented research stream within the Responsible Research and Innovation scholarship, which aims to steer innovations toward the “right impacts” by anticipating their economic, ethical, social, and environmental consequences (35). Inspired by Stilgoe, Owen, and Macnaghten (36), for whom “responsible innovation means taking care of the future through collective stewardship of science and innovation in the present,” RIH defines concrete characteristics through which health technologies may support a more equitable and sustainable pathway for health systems (37).

As Table 2 summarizes, RIH relies on an integrated set of responsibility attributes that fall within five value domains to cover the *processes* leading to innovation (e.g., inclusiveness), the *characteristics* of the innovation (e.g., eco-responsibility), and the for-profit or not-for-profit *organization* that makes it available to end users (e.g., business model) (38). According to these value domains, health technologies should: (i) increase the ability to meet collective needs while tackling health inequalities (population health value); (ii) provide an appropriate response to system-level challenges (health system value); (iii) deliver affordable high-quality products (economic value); (iv) reduce as much as possible their environmental impacts along their lifecycle (environmental value); and (v) be produced by enterprises that strive to provide more value to users, purchasers, and society (organizational value). The overall responsibility of a new technology indeed depends upon the priorities of its manufacturer, an aspect often overlooked in HTAs (39).

**Table 2.** The value domains and attributes of the RIH framework (13) and assessment tool (40). *Only applicable to digital and AI-based solutions (43)

Value domains	Responsibility attributes
Population health value	**Health relevance**: The importance of the health needs addressed within the overall burden of disease, considering the causes of death, injury and disability, and risk factors in the users’ region.
	**Ethical, legal, and social issues (ELSIs):** The means by which the innovation’s negative impacts on the moral and sociocultural well-being of individuals and groups and the legal issues it raises can be mitigated.
	**Health inequalities**: The extent to which avoidable health status differences across individuals and groups associated with one’s socioeconomic status, social position, and capabilities is reduced.
	Human agency*: The means by which a digital solution enables individuals and groups to actively and independently decide and act in accordance with their own goals.
Health system value	**Inclusiveness**: The degree of stakeholder engagement in the design, development, and pilot stages of an innovation using an accountable method.
	**Responsiveness**: The ability to provide dynamic solutions to existing and emerging challenges in health systems (e.g., demographic or epidemiologic shifts, service delivery, or governance gaps).
	**Level and intensity of care**: The optimization of labor intensity that results from mobilizing the most decentralized unit in the health system to provide the service when it is possible to do so effectively and safely.
	Care-centric interoperability*: The extent to which a digital solution securely operates within and across clinical and nonclinical settings without adding significant cognitive and/or administrative burden.
Economic value	**Frugality**: The provision of greater value to more people using fewer resources through a focus on: affordability; core functionalities and ease of use; and optimized performance.
Organizational value	**Business model**: The propensity to provide value to society: social, not-for-profit, or environmental mission; patent-free innovation; redistributive price scheme; employees with particular needs, and so on.
	Data governance*: The transparent and accountable stewardship of the data an organization gathers, exploits, generates, stores, shares with users and third parties (voluntarily or not), and destroys.
Environmental value	**Eco-responsibility**: The reduction of negative environmental impacts along the innovation’s lifecycle stages: raw material sourcing; manufacturing; distribution; use; and disposal.

*Note*: To measure the degree of responsibility of an innovation, the RIH Assessment Tool considers the available evidence and the region where users are located. Each attribute is assessed through a four-level scale, ranging from A to D, where A implies a high degree of responsibility and D has no particular signs of responsibility (which does not mean that the innovation is “irresponsible” but rather signals the absence of this RIH feature).

The RIH Assessment Tool (40), which is among the rare tools that quantitatively measure the degree of responsibility of an innovation, is compatible with HTA practices that assess how a technology *provides value* to the health system in which it is used. For instance, INESSS, which operates in a publicly funded health and social care system, favors collective choices that are “focused on creating value in health care and social services for the benefit of users, patients and their families, and Quebec’s population as a whole” and, for doing so, it “supports responsible innovation for a sustainable development of the health system” (41). Like INESSS’ multidimensional value appraisal framework (42), RIH supports a global value appraisal approach where tensions within and across value domains are carefully documented to clarify the trade-offs at play.

Because RIH highlights the tangible ways in which technologies may generate value in a health and social care system (37), it may help enrich HTA practices in three ways (see Table 3 for brief examples). First, for HTA agencies that rely on a multidimensional value appraisal framework (e.g., the HTA core model), key attributes of the RIH framework can be introduced within the HTA process itself. Second, HTA agencies whose value appraisal process focuses on clinical benefits and costs may use RIH attributes to align their priority-setting or strategic planning activities with system-wide sustainability challenges that are specific to their context. Third, HTA agencies producing early HTAs, horizon scans, or reports that are broader in scope than a single technology review may use the RIH framework to inform their scientific and grey literature search strategy on system-level topics (e.g., coping with drug supply chain disruptions, deploying telehealth services adapted to the needs of individuals living with dementia).

**Table 3.** A summary of how RIH may inform HTA practices

What do RIH and HTA have in common?		
Definition of HTA	Definition of RIH	Implications
“HTA is a multidisciplinary process that uses explicit methods to determine the value of health technology at different points in its lifecycle. The purpose is to inform decision-making to promote an equitable, efficient, and high-quality health system” (65)	“RIH consists of a collaborative endeavor wherein stakeholders are committed to clarify and meet a set of ethical, economic, social and environmental principles, values and requirements when they design, finance, produce, distribute, use and discard sociotechnical solutions to address the needs and challenges of health systems in a sustainable way” (13)	RIH and HTA are both multidisciplinary in scope
		They share a lifecycle approach as well as a concern for equitable and high-performing health systems
		RIH is not a substitute for HTA, it is complementary: RIH’s emphasis on sustainability seeks to reconcile the supply of health innovations with the demand for health systems
How may RIH inform HTA practices?		
Where/when	Aim	Examples
1. Within a multidimensional value appraisal HTA process	To supplement a HTA evaluation process with data describing the value an intervention may bring to (or subtract from) the fulfillment of a health system’s mission	The assessment of a new clinical care pathway that uses the RIH “level and intensity of care” attribute to consider whether the level of care mobilized is safe and efficient
		The assessment of a digital solution that uses the RIH “inclusiveness” attribute to examine whether an accountable method was used by its developer to fulfill clinicians’ needs and increase patient care experience
2. When identifying priority topics or developing a strategic plan for the HTA agency	To align a priority-setting or strategic planning activity with the challenges of the health system in which an HTA agency operates	A priority-setting exercise that uses the RIH “health relevance” attribute to identify the most important unfulfilled health needs within the jurisdiction
		A strategic plan that uses the RIH “eco-responsibility” attribute to introduce environmental considerations in its workplan
3. When producing early HTAs, horizon scans, or reports that are broader in scope than a single technology review	To help decision-makers anticipate the *direction* taken by fast-moving innovations and their impact on health and social care systems	A horizon scan that relies on the RIH “ELSIs” attribute to identify ethical, legal, or regulatory gaps associated with CAR-T cell therapies
		A report that uses the RIH “business model” attribute to describe novel risk-sharing procurement strategies in rare diseases

### Aligning HTA with system-level informational needs

**Figure 2.**
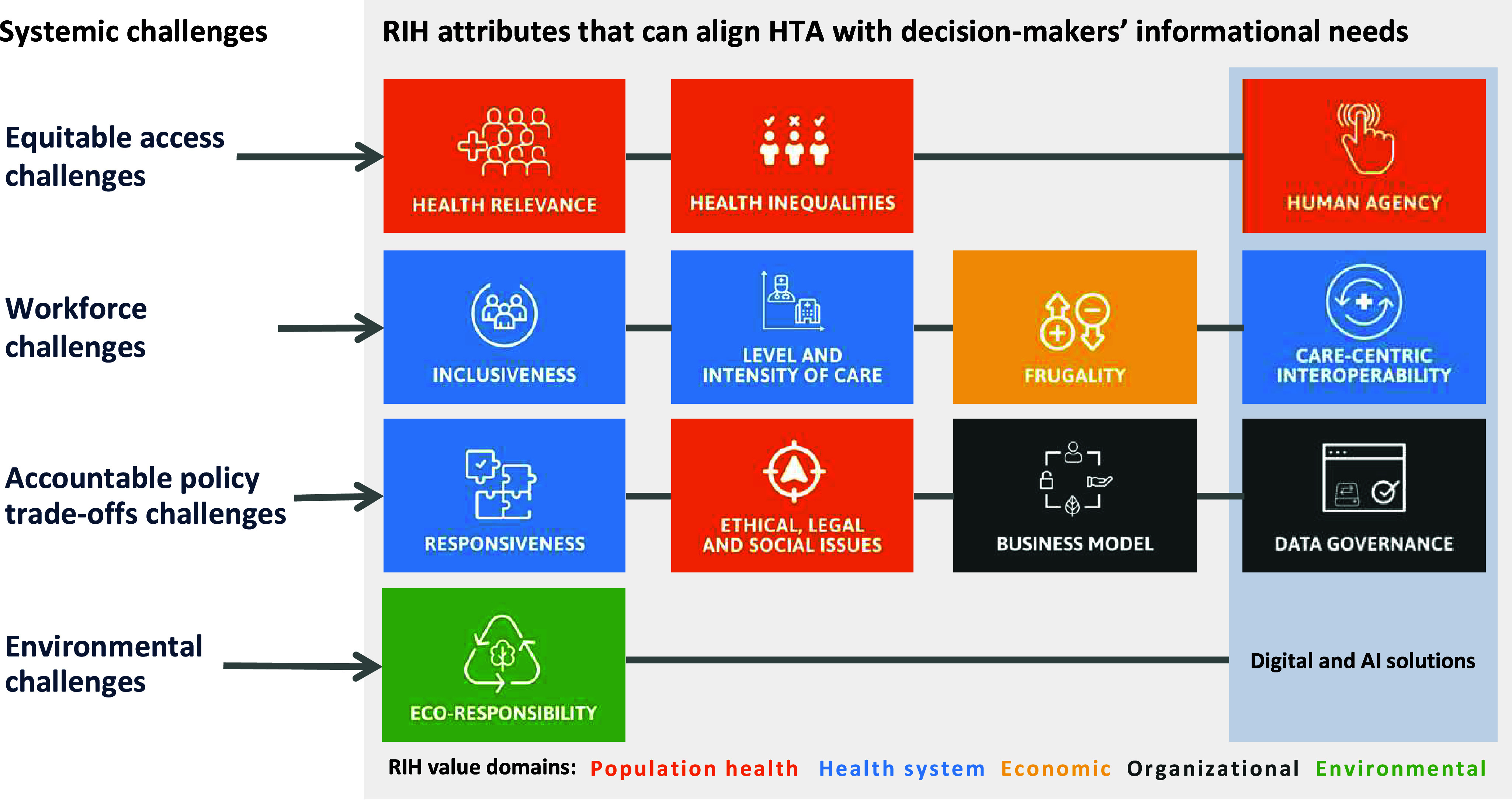
How RIH may support HTA responsiveness to four systemic challenges (see Table 2 for the definition of the attributes). *Source*: the authors.

Figure 2 provides an overview of the responsibility attributes that are part of the RIH framework and can help HTA agencies address decision-makers’ informational needs regarding the four systemic challenges introduced earlier. The shaded box in Figure 2 indicates attributes that specifically apply to digital and AI-based solutions as the latter cut across the four challenges (43).

First, the RIH “health relevance” and “health inequalities” attributes can inform *equitable access challenges.* They focus on the value of technologies that tackle significant unmet needs and deliberately seek to address the health risks to which vulnerable groups are exposed, that cumulate along one’s life course and lead to complex comorbidities. This is aligned with the needs-driven assessments of the International Network for Social Intervention Assessment (INSIA) and the checklist developed by Benkhalti, Espinoza (44) to guide equity considerations in HTA. These authors stress that health inequities result from social determinants of health such as “place of residence, race/ethnicity/culture/language, occupation, gender/sex, religion, education, socioeconomic status, and social capital” (see PROGRESS-plus of the Cochrane Equity Method Group (45)). Notwithstanding the lessons learned from the COVID-19 pandemic regarding “longstanding social disparities and issues of discrimination, racism, and inequitable access to care” (46), such considerations are at the core of RIH and can inform HTA agencies’ priority-setting or strategic planning activities. When it comes to digital and AI-based solutions, “human agency” stresses the need to examine the extent to which they enable patients and/or healthcare professionals to independently decide and act in accordance with their goals (43). Here, integrating multiple sources of evidence, a practice already in place at INESSS (41), can document the practical experiences of frontline practitioners, patients, and their caregivers, including those who lack digital infrastructures or literacy (47;48).

Second, four RIH attributes shed light over systemic *workforce issues*, such as staff recruitment, distribution, retention, and shortages, as they focus on the value of technologies that are carefully designed to support health and social care providers’ tasks. RIH recognizes that value intimately lies with a technology’s ability to consolidate the skills and range of actions of its users, including community and social service providers. As a result, “inclusiveness” examines whether technology development processes have gathered and responded to the needs of a broad range of users, and “level and intensity of care” follows the subsidiarity principle according to which the lowest level of care should be mobilized to deliver a service when it is possible to do so safely and effectively (28). “Frugality” values interventions that are not only affordable, but also easy to use by a greater number of individuals and optimized for varying contexts of use (e.g., rural, or remote areas) (49). Taken together, these three attributes can help HTA practitioners appraise the value of technologies that are responsive to the context and skills of healthcare workers at different levels in the health system. “Care-centric interoperability” helps identify whether a digital solution can securely operate within and across care settings (i.e., following the patient along the clinical care pathway) without adding significant cognitive and/or administrative burden to users. It is informed by growing evidence on the way computerized medical records and other digital platforms contribute to professional exhaustion, which then affects the quality of care (50). In areas such as juvenile delinquency, mental health, elderly care, and disability care (51), the health outcomes that matter in HTAs are particularly sensitive to the availability of human resources. Gathering evidence on the four attributes can clarify whether and how technology supports health and social care practitioners in their daily tasks as well as quality and continuity of care for patients (32).

Third, as HTA agencies are being asked to go beyond traditional methods to support “implementation into policy and clinical practice” (17), four RIH attributes can help document *policy trade-offs challenges.* The “ELSIs” attribute examines whether adequate means are available to mitigate the ethical, legal, and/or social issues a given technology may raise. Such means may include, for instance, user-friendly patient decision aids, proper post-market surveillance, or community programs to reduce social stigmatization. Knowing that adequate tools are in place may reduce uncertainty in HTAs where contextual factors affect the outcomes of a technology (52). HTA horizon scans may also bring to policymakers’ attention regulatory gaps that put patients at risk. For example, during the COVID-19 pandemic, the US Food and Drug Agency quickly set in place regulatory relief for apps addressing depression, anxiety, and insomnia, even if little evidence was available to support their use (48). “Responsiveness” identifies the type of systemic challenges a technology addresses (e.g., service delivery gaps, coordination across care providers) as well as its level of importance for the health system (i.e., how high it ranks among documented priorities). Two RIH attributes can help flag acquisition and procurement issues, ranging from medical supplies to Software as a Service (SaaS) licenses. “Business model” highlights whether a manufacturer operates a shareholder- or a stakeholder-centered business model, the latter being more likely to adhere to a sustainable triple bottom line that can align with the mission of health systems. “Data governance” examines whether digital tech companies have accountable mechanisms to ensure the quality and control of the entire lifecycle of the data associated with their solutions. This policy concern will become increasingly important in HTA as digital health solutions keep spreading within and outside formal regulatory approval pathways (47). Providing clear signals to this rapidly growing yet unstructured industry through stakeholder dialogues, for instance, can allow scientific guidance “to be delivered to multiple technology manufacturers at once” more efficiently (4).

Finally, the *environmental challenges* that health systems face will require extensive efforts, both in terms of research and practice (53;54). The RIH “eco-responsibility” attribute represents a small step, but one that aligns with recent work in HTA (55). For Polisena, De Angelis (26), the environmental assessment of a health technology should consider its entire lifecycle, from raw material sourcing to proper end-of-life, and this is the perspective adopted in RIH. Other approaches involve examining environmental harms to human health from an economic perspective (e.g., DALYs) (34). In their scoping review of articles and guidelines that jointly addressed environmental and economic dimensions, Desterbecq and Tubeuf (25) observed that Canada’s Drug Agency and the HTA unit of the Ministry of Health in Brazil had “included environmental impact as a relevant criterion in their economic evaluation guidelines.” Twenty-seven percent of the documents retrieved originated from the United Kingdom and this was seen as consistent with the proactive Net Zero 2040 agenda of the National Health Service, which prompted a public consultation by the National Institute of Clinical Excellence (NICE) (56). Decision-makers will increasingly ask guidance from HTA agencies to support such systemic change as the COP26 climate-smart health program endorsed by fifty countries urged them to reduce healthcare’s environmental footprint (57).

To summarize, though making investment and disinvestment trade-offs remain under decision-makers’ responsibilities (46), RIH offers a framework that can help HTA agencies to clarify how health technologies may tangibly add (or subtract) value by affecting interconnected system-wide challenges.

## Practical implications

This article may offer food for thought in a period where HTA agencies and health system leaders are reconsidering their priorities (17). At INESSS, when we explored how RIH may enrich our practices, we identified three practical implications. These may inspire other HTA agencies to increase their responsiveness to decision-makers’ informational needs regarding system-wide challenges, keeping in mind that the RIH attributes summarized in Table 2 may be used by HTA agencies to: (i) supplement their evaluation process; (ii) align their priority-setting or strategic planning activities with the challenges of the health system in which they operate; or (iii) inform the production of early HTAs, horizon scans, or reports that are broader in scope than a single technology review (see specific examples in Table 3).

At INESSS, a first practical implication that had immediate relevance to our agency, which already does substantial work on the optimal use of screening and diagnostic tests, imaging devices, and drugs, was to foreground in our 2024 – 2028 strategic plan the appraisal of interventions sitting at the “intersection” of healthcare overuse and environmental harms (34). Because overdiagnosis and overtreatment may unnecessarily expose the patient to harm, increase clinical workload, and represent “wasteful” spending (58), reducing low-value care represents a systemic lever to decrease “healthcare emissions and pollution, without compromising health outcomes” (34). This strategy thus targets positive synergies (or “co-benefits”) in the mitigation of health system challenges and is aligned with the responsible procurement framework of Quebec’s Ministry of Health and Social Services (MSSS) (59).

Second, INESSS has drawn on RIH to consolidate its early HTA practices in collaboration with Quebec’s health innovation ecosystem stakeholders, including the Innovation Bureau of the MSSS, academic health centers, hospital-based HTA units, and the network of innovation respondents. This is an area where uncertainty prevails and where multidisciplinary communication skills are of utmost importance. Uncertainty in HTA may have to do with “the relevance, completeness, and trustworthiness of data” (16). It calls for improved communication because different groups must make sense of the evidence that is available when technology remains immature and of the evidence that will be needed later to decide whether to support its deployment and in which contexts. We have thus engaged multiple groups in the development of a user-friendly early HTA lexicon and a practical guide to global value appraisal (60). Articulating the “full chain of reasoning” underpinning HTAs is necessary for transparency (19) but it is equally important to apply consistent and easy-to-grasp value appraisal criteria throughout the (re)assessments that may take place along a technology’s lifecycle.

Third, INESSS is redefining the basis of its collaboration with university research centers because a RIH system-wide perspective suggests that the strengthening of the health and social care system requires distinct yet synergistic evidence-based decisions (22;29). Evidence-generation strategies where both expertise and workloads are shared can be built on the recognition that in complex systems “small inputs may have large effects and vice versa” (16). The challenge is thus to prioritize and properly orchestrate the production of evidence tailored to different decision-makers’ needs. As van der Wilt and Oortwijn (61) underscore, HTA agencies and universities can work together to consolidate the policy relevance of specific HTAs. Doing so at a broader scale seems particularly justified when the level of complexity faced by decision-makers calls for a systemic learning process grounded in real-world situations where all can learn to work *with* complexity (16).

## Conclusion

The direction taken by health technologies since the 1990s has created an “increasing demand for HTA and pressure for rapid assessments” (17). Nonetheless, HTA bodies’ capacity to fully support decisions toward equitable and sustainable health systems has not been fully developed. In the near future, many countries will keep grappling with the complex chronic care needs of their populations that will be compounded by acute care needs because of extreme weather events (24), fragmentation in service delivery, workforce shortages, limited budgets, and persistent “gaps between evidence, policy, and practice” (14). RIH offers an integrated lens for HTA agencies to reflect on these system-wide challenges and provide forward-looking guidance. HTA agencies hold a unique set of skills that can be mobilized to help decision-makers make health systems more successful and sustainable. We thus concur with McGurn (46), for whom “finding the path forward” amid current uncertainty is both inevitable and enlivening.
